# Older people’s perceived autonomy in residential care: An integrative review

**DOI:** 10.1177/0969733020948115

**Published:** 2020-10-01

**Authors:** Tanja Moilanen, Mari Kangasniemi, Oili Papinaho, Mari Mynttinen, Helena Siipi, Sakari Suominen, Riitta Suhonen

**Affiliations:** 8058 University of Turku, Finland; 8058 University of Turku, Finland; Oulu University Hospital, Finland; 8058 University of Turku, Finland; 8058 University of Turku, Finland; Turku University Hospital, Finland; City of Turku Welfare Division, Finland

**Keywords:** Autonomy, care professionals, integrative review, older people, residential care, self-determination

## Abstract

Autonomy has been recognised as a key principle in healthcare, but we still need to develop a consistent understanding of older people’s perceived autonomy in residential care. This study aimed to identify, describe and synthesise previous studies on the perceived autonomy of older people in residential care. Ethical approval was not required, as this was a review of published literature. We carried out an integrative review to synthesise previous knowledge published in peer-review journals in English up to September 2019. Electronic and manual searches were conducted using the CINAHL, Philosopher’s Index, PubMed, SocINDEX, Scopus and Web of Science databases. The data were analysed using the constant comparison method. The review identified 46 studies. Perceived autonomy referred to the opportunities that older people had to make their own choices about their daily life in residential care, and achieving autonomy promoted both health and quality of life. Autonomy was linked to older people’s individual capacities, including their level of independence, physical and mental competence, personal characteristics, and whether relatives shared and supported their perceived autonomy. Professionals could facilitate or hinder older peoples’ autonomy in a number of ways, including providing opportunities for autonomy, how daily care needs and activities were managed, and controlling older people’s choices. Professionals’ characteristics, such as education and attitudes, and the older people’s living environments were also associated with their perceived autonomy and included organisational characteristics and physical and social care facilitators. Older people’s perceived autonomy promoted health and quality of life in residential care. However, their autonomy was associated with a number of protective and restrictive individual and environmental factors, which influenced whether autonomy was achieved.

## Introduction

Organisations that provide residential care for older people should make sure that they enjoy their daily lives,^1^ and respecting their autonomy is an important part of that. Autonomy refers to older people’s rights to make decisions without being influenced by others,^2,3^ and it is linked to older people’s abilities and opportunities to govern themselves. Perceived autonomy is older people’s individual perceptions of their abilities and opportunities related to autonomy. Older people have reported that perceived autonomy increased their quality of life and satisfaction with daily routines in residential care.^4^ It has also been associated with improved health and well-being^5^ and resulted in organisational benefits, such as increased staff retention.^4^

It has been recognised that living in residential care can influence how older people perceive their autonomy.^6^ Ageing has an impact on an individual’s health status^7^ and their physical ability to engage in daily activities.^8,9^ Autonomous decision-making requires sufficient knowledge, skills and abilities,^10^ together with resilience, a sense of self-efficacy and material opportunities, such as financial independence.^7^

Autonomy has been identified as one of the basic ethical principles of healthcare,^11^ and it is a human right that is protected by universal declarations and conventions.^12,13^ Although autonomy is an inherent basic human right, individuals perceive autonomy differently and its meaning can also vary depending on the time and context. Perceived autonomy is crucial for older people, because their ability to assert their autonomy can be reduced by being admitted to residential care^6^ and having to depend on others.^10,14^ This puts them in a vulnerable position.^10^ Research has shown that older people have suffered from paternalism and a lack of opportunity to participate in decisions about themselves.^9^ That is why autonomy is a core value in older people’s care.^15^ Professionals need to be aware of what autonomy means and they need to consider how older people perceive autonomy as part of their everyday practice.^12,16^ Professionals face wide-ranging responsibilities when it comes to recognising and enabling older people’s perceived autonomy in residential care. This can be challenging, because previous research has failed to establish a consistent understanding of the perceived autonomy of older people.

## Aim

This study aimed to identify, describe and synthesise previous studies on older people’s perceptions of autonomy in residential care. We wanted to find out how this had been described in previous studies and what factors had supported or hindered it.

## Method

We employed the integrative review method, as this enabled us to synthesise studies that were produced using different methods.^17^

### Literature searches

**Figure 1.**
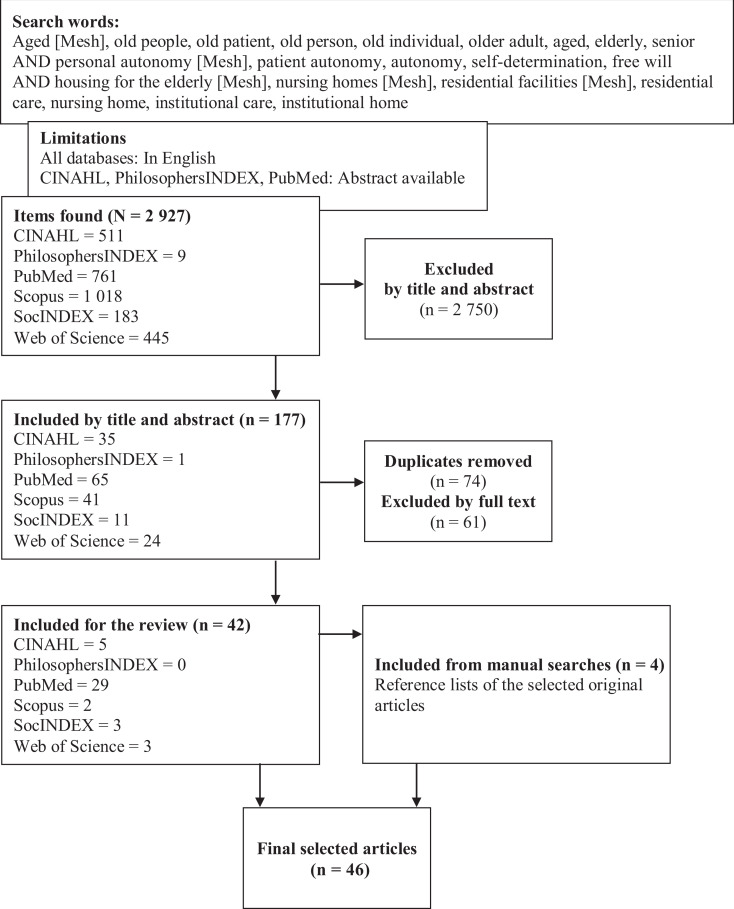
Flowchart of literature searches.

We conducted preliminary searches of existing studies to identify the most eligible search terms and their combinations. This was done in collaboration with an informatics specialist. Electronic searches were conducted using the CINAHL, Philosopher’s Index, PubMed, SocINDEX, Scopus and Web of Science databases. We used Boolean operators and combinations of MeSH terms and free search terms, with their synonyms, to identify studies that covered older people, autonomy and residential care (Figure 1). Manual searches were also carried out, by scrutinising the reference lists of the selected papers. The limitations for the searches were that the article had to be published in English in a peer-reviewed journal and that an abstract was available. All results up to September 2019 were assessed.

### Search outcomes and retrieval

The selection was based on the inclusion and exclusion criteria established by the Sample, Phenomenon of Interest, Design, Evaluation, Research tool for synthesising previous evidence (SPIDER)^18^ (Table 1). We included empirical studies that focussed on the autonomy, self-determination or free will of older people living in long-term care. Literature reviews, theoretical papers and other papers, such as editorials and commentaries, were excluded.

**Table 1.** SPIDER^18^ inclusion and exclusion criteria.

SPIDER	Specifications
Sample	Older people in residential care
Phenomenon of interest	Autonomy
Design	Empirical study
Evaluation	Reported outcomes/results
Research type	Scientific, qualitative, quantitative or mixed-method study

The electronic searches yielded 2927 results: 177 original papers were selected based on their title and abstract and 42 on their full text. The manual searches identified 10 potential papers, based on their title, and 4 were selected for the final review. A total of 46 original papers were independently selected by two authors (TM and MK) based on the searches. Any potential disagreements about the selected papers were discussed and the final selection was based on consensus.

### Data evaluation

The quality of the selected papers was evaluated using the Mixed Methods Appraisal Tool evaluation criteria (MMAT).^19^ The maximum score for the quantitative and qualitative studies was six and it was five for the mixed methods and randomised controlled trials. The evaluation, which was independently conducted by two researchers (TM and MK), aimed to describe the methodological quality of the original papers (Table 2). The quality of the selected papers was high. There were some missing values in the quantitative studies about the response rates and qualitative studies’ considerations of the researchers’ influence.

**Table 2.** Summary of the selected studies.

Author(s), country	Aim	Method, data and care facility	Concept(s)	Main results	Quality score
Ryden,^20^ United States	To delineate characteristics of residents’ interpersonal, organisational and physical aspects of autonomy	Semi-structured questionnaire for older people (n = 113), professionals (n = 137) and managers (n = 10)	Autonomy	Older people had the least control with regard to the everyday issues of grooming and eating in the facility.	5/6
Saup,^21^ Country not stated	To examine how older people experienced lack of autonomy and stress and how they coped with them	Survey of older people (n = 35)	Autonomy	Older people’s coping strategies for stress varied in relation to how the facility emphasised personal autonomy.	5/6
Grow Kasser and Ryan,^22^ United States	To examine how personal autonomy and social support were correlated	Survey of older people (n = 50)	Autonomy, Self-determination	Autonomous self-regulation was positively correlated with subjective health. Social support correlated positively with autonomy.	6/6
Barkay and Tabak,^23^ Israel	To describe autonomy and participation experienced by older people in institutions	Survey of older people (n = 39)	Autonomy	Older peoples’ autonomy positively influenced social functioning and satisfaction with life.	6/6
Scott et al.,^24^ United Kingdom	To examine older people’s views about autonomy, privacy and informed consent	Survey design with structured interviews with older people (n = 101) Self-completed questionnaire for professionals (n = 160)	Autonomy	Only 15% of older people reported that professionals had always given them information during some treatment procedures. Older people who were more educated received more often information from professionals than others. According to the older people, professionals did not provide them with opportunities to make decisions about pain relief methods.	5/6
Scott et al.,^25^ Finland, Germany, Greece, Spain, United Kingdom	To examine autonomy in healthcare institutions in European countries	Survey design with structured interviews with older people (n = 573) Self-completion questionnaire for professionals (n = 887)	Autonomy	Opportunities to make decisions varied from country to country. They were highest in Spain and lowest in Germany.	2/6
Boyle,^26^ Ireland	To explore older people’s experience about whether they had choice and control over the support they received	Structured surveys (n = 214) and interviews (n = 45) with older people	Autonomy	Older people in institutions had greater autonomy to make decisions than people cared for in their own homes.	5/6
Boyle,^27^ United Kingdom	To examine the extent of depression and mental health among older people in long-term care	Structured interviews with residents in an institution (n = 214) and older people receiving care in their own homes (n = 44)	Autonomy	Older people in institutions seemed to have higher perceived opportunities for their own choices than people who received care in their own homes. Older people with poor mental health perceived fewer choices.	5/6
Hwang et al.,^28^ Taiwan	To explore factors related to older people’s autonomy	Questionnaires for people living in a home for senior citizens (n = 121)	Autonomy	Older people’s autonomy was affected by social support, functional abilities, attitudes and their educational level.	5/6
Sikorska-Simmons,^29^ United States	To explore how organisational policies affected residents’ perceptions of autonomy in assisted living	Questionnaires for residents in facilities (n = 412)	Autonomy	Choice-enhancing policies improved older people’s experience of autonomy in assisted living facilities.	6/6
Andersson et al.,^30^ Sweden	To describe older people’s experiences of daily life in a care home	Interviews with residents (n = 13), relatives (n = 10) and contact persons (n = 11)	Self-determination	Older people felt that their self-determination was unsatisfactory in care homes.	5/6
Boisaubin et al.,^31^ United States	To explore perceptions of autonomy, dignity, quality of care and decision-making	Pilot study with semi-structured interviews with older people in long-term care (n = 4), family members (n = 10) and healthcare professionals (n = 9)	Autonomy	Older people felt that they should keep their authority and responsibility for decision-making as long as possible. When they were no longer capable, then relatives should make decisions for them.	4/6
Hellström and Sarvimäki,^32^ Sweden	To describe how older people in sheltered housing experienced self-determination	Interviews with residents (n = 11)	Self-determination	Older people had negative experiences about how their self-determination was acknowledged in the facility and how the environment did not support their self-determination.	5/6
Chan and Pang,^33^ China (Hong Kong)	To examine perceptions about individual dignity and autonomy, quality of care and financing of long-term care	In-depth semi-structured interviews with older people (n = 6), family members (n = 10), administrators (n = 6) and healthcare professionals (n = 7)	Autonomy	Older people were unanimous, that they should be allowed to make final decisions. They were concerned about whether their wishes and needs were taken into account. Older people’s decisions were often compromised by relatives’ opinions.	5/6
Zhai and Qiu,^34^ China	To study perceptions about long-term care for older people	Descriptive interviews with older people (n = 6), relatives (n = 10), assistant nurses (n = 4), physicians (n = 3) and managers (n = 3)	Autonomy	Older people felt that their autonomy should be respected, but this did not mean that the older person was the only decision-maker.	3/6
Andresen et al.,^35^ Denmark	To evaluate individually tailored programmes on autonomy for institutionalised older people	Blinded randomised trial for older people (n = 50)	Autonomy	Individualised support can enable institutionalised older people’s autonomy.	5/5
Hawkins et al.,^36^ United Kingdom	To examine how healthcare workers supported residents’ autonomy by enabling their independence	Ethnographic approach, with qualitative observations, semi-structured interviews (14 staff members and 8 older residents), documentary analysis	Autonomy	Older people’s autonomy was supported by enabling independence. However, conflicts between the staff’s duty to care and support autonomy was acknowledged.	6/6
Nakrem et al.,^37^ Norway	To describe residents’ experiences of their nursing home and the quality of care they received	Descriptive, exploratory design, in-depth interviews with older people (n = 15)	Self-determination	Older people recognised it was challenging to balance self-determination and dependency. Feelings of indignity and depreciation of social status were reported.	6/6
Custers et al.,^38^ Netherlands	To examine older people’s experiences of relatedness, autonomy and competence in the caring relationship. The social nature of human beings and how they connect	Questionnaire (n = 75) and semi-structured interviews with older people (n = 35)	Autonomy, self-determination	Relatedness was perceived to be more important than autonomy and competence. Subjective health and cognitive functioning were correlated with relatedness, autonomy and competence.	5/6
Candela et al.,^39^ Italy	To identify the links between autonomy and daily living activities in institutionalised older people	Survey of older people (n = 40)	Autonomy	Older people seemed to have sufficient cognitive abilities to maintain their autonomy. Older people who had low levels of perception of their physical functioning had low levels of autonomy.	5/6
Riedl et al.,^40^ Austria	To examine what nursing home residents needed when they moved to nursing homes	Interviews with older people (n = 20)	Self-determination	Older people said that decisions were taken away from them and that they needed to fight for their independence.	5/6
Wulff et al.,^41^ Germany	To investigate perceived autonomy of nursing home residents	Survey of older people (n = 560)	Autonomy	Older people chose when they wanted to be active in decision-making and when they preferred to leave the decisions for others. Their health status restricted their opportunities for autonomous decisions.	5/6
Cobo,^42^ Spain	To examine the influence of institutionalisation on autonomy and quality of life	Quasi-experimental and longitudinal survey of older people (n = 104), lasting 20 months	Autonomy	The relationship between older people’s autonomy and independence was identified and both factors deteriorated due to institutionalisation.	5/6
Ferrand et al.,^43^ France	To examine older people’s psychological satisfaction and the relationship between that and well-being	Survey of older people (n = 100)	Self-determination	Older people’s autonomy was linked to their satisfaction with their need for relatedness and their purpose in life. Autonomy and satisfaction with their need for relatedness were identified as indicators of well-being.	5/6
Hillcoat-Nallétamby,^44^ United Kingdom	To examine older people’s understanding of independence and autonomy in a residential setting	Interviews with older people (n = 91)	Autonomy	Older people linked independence to accepting help and doing things alone. Delegated, executional, authentic, decisional and client autonomy were identified.	6/6
Taverna et al.,^45^ United States	To examine the effect of autonomy on residents’ oral hygiene	Mixed-method approach, with individual structured interviews and open ended and Likert-type scale questions for older people (n = 12) and professionals (n = 7)	Autonomy	Older people’s autonomy was used to resist professional assistants taking charge of their oral hygiene.	4/5
Wikström and Emilsson,^46^ Sweden	To explore opportunities for autonomy in institution-based housing	Focus group interviews (n = 50), observations on residents (n = 17), family members (n = 10), staff (n = 12) and managers (n = 11)	Autonomy	Older people’s ability to maintain control over their lives was influenced by ambivalent missions, symbolic power and professionals’ ageist approaches.	5/6
Chang,^47^ Taiwan	To examine how self-determination and social support with regard to leisure activities were related to stress	Face-to-face surveys for nursing home residents (n = 141) and older people supported in their own homes (n = 322)	Self-determination	Older peoples’ self-determination and social support with regard to leisure choices were negatively linked with stress.	5/6
Ayalon,^48^ Israel	To address older adults’ experiences of the transition to continuing care in a retirement community	Interviews with older people (n = 32) and their relatives (n = 19), older people and adult children dyads (n = 34)	Autonomy	Older people’s autonomy was demonstrated by their ability to exercise independent decisions and a degree of capacity.	5/6
Bollig et al.,^49^ Norway	To study how residents and their relatives perceived ethical challenges in nursing homes	In-depth interviews with older people (n = 25), focus groups with relatives (n = 18)	Autonomy. self-determination	Older people’s experiences of autonomy varied between individuals. Some older people felt that they could make their own decisions, while others felt that they did have not much to decide.	6/6
Donnelly and MacEntee,^50^ Canada	To explore how residents adapted to care and felt about it	Observations and interviews with older people (n = 23)	Autonomy	Older people reported violations of their autonomy and identified poor care practices that threatened their dignity.	5/6
Gjerberg et al.,^51^ Norway	To explore coercion in a nursing home from the point of view of older patients and their family members	Individual interviews with older people (n = 35) and focus groups for relatives (n = 60)	Autonomy	Half of the respondents accepted the use of coercion and believed that the professionals acted in the best interest of the patient.	4/6
Nord,^52^ Sweden	To examine free choice among older people in residential care	Interviews with older people (n = 13)	Self-determination	Older people’s networks made their choices possible. Older people had high autonomy levels about decisions to move in residential care.	4/6
Souesme et al.,^53^ France	To examine older people’s perceptions of support for autonomy and depressive symptoms	Surveys of older people (n = 100)	Autonomy, Self-determination	Low social support for autonomy correlated with depressive symptoms and apathy.	5/6
Tuominen et al.,^54^ Finland	To describe older people’s experiences of free will in nursing homes	Interviews with older people (n = 15)	Free will	Older people saw free will as the opportunity to make their own decisions and maintain their rights. Free will was focused on daily activities. Barriers to free will were their dependency on care, other residents, professionals and institutional rules.	6/6
Walker and Paliadelis,^55^ Australia	To investigate older people’s experiences of living in residential care	In-depth interviews with older people (n = 18)	Autonomy	Older people were excluded from decision-making and this meant that they found it difficult to uphold their autonomy.	5/6
Bennett et al.,^56^ United States	To explore how having visitors affected residents’ autonomy in assisted living	Ethnographic study with observations and informal, in-depth and open-ended interviews with older people (n = 68), relatives (n = 47), professionals (n = 65) and managers (n = 18)	Autonomy	Older people struggled to maintain their former social networks. The facility’s rules and restrictions made social visits complicated and their visitors were mainly family members.	5/6
Cho et al.,^57^ South Korea	To explore older people’s perceptions of their daily lives in nursing homes	Semi-structured individual interviews with older people (n = 21)	Autonomy	Older people experienced limited autonomy, which was linked to quality of life and homelike environments.	5/6
Paque et al.,^58^ Belgium	To examine nursing home residents’ autonomy, social functioning and social environment	Structured questionnaire for older people (n = 391)	Autonomy	Older people had more autonomy when they were able to perform daily living activities independently, than those who had limited capacity and struggled without assistance. Older people with depressive feelings also had limited autonomy.	5/6
Caspari et al.,^59^ Denmark, Sweden and Norway	To examine older people’s experiences of freedom in nursing homes	Interviews with older people (n = 28) in Denmark, Sweden and Norway	Freedom	Older people emphasised the importance of their autonomy and having their dignity acknowledged. However, older people felt that their care dependence and the paternalistic attitude displayed by professionals were barriers to their freedom.	6/6
Chang,^60^ Taiwan	To examine the effects of self-determination and social support with regard to leisure on stress	Face-to-face survey of older people (n = 139)	Self-determination	Being able to choose leisure activities was negatively correlated with stress.	6/6
Lin and Yen,^61^ Taiwan	To assess the relationship between leisure activities and adjustment to residential care	Survey of older people in long-term care (n = 136)	Autonomy	Taking part in leisure activities helped older people to gain autonomy and control how they adjusted to residential care.	6/6
Tufford et al.,^62^ Canada, Germany, Norway and United Kingdom	To examine locked door practices and how they were justified in long-term care facilities	Observations of, and interviews with, older people, relatives and professionals (n = 285)	Autonomy	Older people’s ability to move within and outside facilities depended on the locked door practices and accessibility. Lock doors could also provide security for older people.	4/6
Buckinx et al.,^63^ Belgium	To evaluate isometric strength levels on autonomy among nursing home residents	Survey of older people (n = 662)	Autonomy	Being unable to use knee and ankle extensors independently predicted loss of autonomy.	5/6
Kloos et al.,^64^ Netherlands	To examine relationships between subjective well-being and satisfaction with autonomy, relatedness and competence	Questionnaire for older people (n = 128)	Self-determination	Older people’s autonomy was associated with life satisfaction and loss of autonomy was associated with depressive feelings.	6/6
Schenell et al.,^65^ Sweden	To examine perceptions of quality of care and self-determination	Cross-sectional study with questionnaire for older people (n = 112) and relatives (n = 83)	Self-determination	Older people wanted to keep their decision-making power for themselves and seldom wanted to hand it over to relatives or professionals.	6/6

### Data analysis

The analysis began by reading all the selected papers to provide an overview of the content. The methods used by the papers were tabulated according to the publication, the aim of the study, the methods used and the study concepts (Table 2). Descriptions of perceived autonomy, such as words, sentences or phrases, were identified and coded. Then the material that focused on older people’s autonomy was extracted and analysed by the constant comparison method. This means that the extracted expressions from the individual papers were grouped based on their similarities and differences. They were also compared to other papers, and the entire results, at the same time, to ensure that the analysis was representative and not biased.^66,67^ No interpretation was carried out and the original text was used for the analysis.

## Results

### Descriptive information about the studies reviewed

The 46 studies that we reviewed were published between 1985 and 2019. Of these, 24 were quantitative, 21 were qualitative and there was one mixed-method study. All of the studies sought the views of older people: 11 also spoke to professionals^20,24,25,31,33,34,36,45,46,56,62^ and 11 studies also included feedback from relatives.^30,31,33,34,46,48,49,51,56,62,65^ The studies used concepts such as autonomy (n = 34), self-determination (n = 14), free will (n = 1) and freedom (n = 1). Most of the studies were conducted in Europe (n = 30), with seven in North America, seven in Asia and one in Australia. One study did not specify the country (Table 2). This review focused exclusively on the views expressed by older people and did not include feedback from care professionals and relatives.

### Perceived autonomy of older people in residential care

Older people’s autonomy was described as exercising their own free will^48,54,56^ and their ability to make independent choices,^26,31,44,48,52,56,59^ without domination or suppression.^54^ Their autonomy related to decisions about nutrition,^37,46,49,54^ rest and sleep,^26,54,58,59^ outdoor activities^40,59^ and hygiene,^26,37^ including clothing.^26,58^ Older people wanted to make their own decisions about their own social activities^41,58^ and receiving visitors.^44,56,58^ Perceived autonomy also included how they used their money^31^ and decorated their own room.^37,44,52^

Autonomy was considered as a sign of respect,^31^ dignity^30,33,59^ and human value^32^ and how they were regarded as an individual.^32,34,44^ Older people said that their autonomy promoted their well-being,^64^ subjective vitality^22^ and mental health.^27^ They also felt that it decreased stress levels,^21,37,60^ depression and apathy,^22,27,53,64^ and improved their quality of life and satisfaction.^33,43,64^ Those who were satisfied with their autonomy were also more active and satisfied with the activities provided by their residential care home.^23^ In contrast, limited autonomy led to feelings of confinement and frustration^26,32,57^ and increased the overall mortality rate.^22^

### Factors supporting and hindering perceived autonomy among older people

Older people felt that their autonomy in residential care was associated with their own individual capacity, how they saw the care practices provided by professionals and the living environment provided by the residential care home (Table 3).

**Table 3.** Factors supporting and hindering perceived autonomy among older people.

Main category	Supporting and hindering factors
Older people’s individual capacities	*Older people’s competence*
	Capacity to act^30,39,44,48,58^
	Increased need of care^32,37^
	Stress, low energy level, social isolation^58^
	*Personal characteristics*
	Education level^28^
	Financial abilities^32,44,48,59^
	*Relatives sharing and supporting autonomy*
	Respect older people’s decisions^34^
	Involvement of relatives^31,33,34,44,52^
	Pressure to make decisions^48^
Professionals facilitating perceived autonomy	*Professionals’ activities to create opportunities for perceived autonomy*
	Support from professionals for perceived autonomy^22,28,60^
	Respect for older people’s autonomy^32,34,49,59,66^
	Recognition of older people’s will^33,35,54^
	Advance directives^31,33^
	Information giving^20,24,25,32,46^
	*Professionals’ controlling choices*
	Professionals make decisions on behalf of older people^25–27,32,36,37,50,55^
	Unethical conduct^54^ and coercion^49^
	Allowing opportunity to complain^32,46,50,54,59^
	*Enabling care practices*
	Care routines^21,26,27,32,36,38,49,59^
	Availability of assistance^32,44,49,54,59^
	*Professionals’ characteristics*
	Professionals’ education^32,45,54^
	Flexibility,^54,63^ equality, positivity,^54^ friendliness^38^
	Arbitrary, manipulative or power-seeking attitude^54^
Living environment creating opportunities	*Organisational characteristics*
	Involvement of older people^20,29,54^
	Group focused rules^57^
	Type and size of facility^26,27,32,36,54^
	Limited resources^26,32,45,49,50,54^
	*Physical care facilitators*
	Easily accessible care environment^20,37,40,54,57,59,62^
	Locked doors^49,59,62^
	*Social care facilitators*
	Privacy^20,24,59,62^
	Other residents^46,54^
	Participation in social activities^32,36,46,54,61^
	Visits from friends and realtives^22,23,52,56^

#### Older people’s individual capacities

##### Older people’s competence

Older people felt that expressing their own opinions and maintaining their rights^20^ would enhance their autonomy.^29,54^ They also said that their level of independence allowed them to make autonomous decisions^27,28,44,54,58^ and improved their opportunities to demand their rights.^54^ Independence referred to the capacity to act,^30,39,44,48,58^ with sufficient physical,^26,28,63^ cognitive^26^ and psychological abilities.^39^ It also included health status and medical conditions.^32,36,38,52,54^ The studies reported that the capacity of older people deteriorated after they were moved to residential care,^42,48^ while the amount of help they needed increased.^42^ Older people who felt they had low competence also perceived low autonomy.^39^ An increased need for care^32,37^ threatened older people’s autonomy, because of their increasing dependency on professionals.^32,37,59^ This increased reliance on others also increased the risk of them being subjected to paternalistic behaviour. Older people said that their decreased functional abilities hindered their physical opportunities, but not their inner freedom to think or react.^59^ They felt that they could keep their autonomy as long as they have sufficient intellectual skills for decision-making.^31^ In addition, older people acknowledged that stress, low energy levels and social isolation hindered their perceived autonomy.^58^

##### Personal characteristics

Older people perceived that their autonomy in residential care was influenced by their educational level and financial abilities: the higher their educational level^28^ and financial abilities, the more autonomy they perceived. Having a better economic background gave them more choice,^44,48,59^ more control over their life^44^ and the ability to acquire more help in residential care.^32,44^ However, age and gender did not seem to influence older people’s perceived autonomy.^32,35,43,44^

##### Relatives sharing and supporting autonomy

Older people said that their relatives influenced their autonomy, but they preferred to make their own decisions,^31,33^ which they expected their relatives to respect.^34^ Some older people involved their relatives in decision-making and shared the burden of decision-making, especially when their capacity to make own choices declined.^31,33,34,44,52^ Other older people preferred to leave any decisions to their relatives,^52,65^ but they expected them to promote and protect their rights.^32,54^ When relatives and friends supported their perceived autonomy, this correlated with positive well-being, vitality, satisfaction with their lives^22^ and decreased depression.^22,53^ However, relatives could also hinder older people’s perceived autonomy, by deciding who could access and visit them^56^ or putting pressure on older people to make decisions in line with their own views.^48^

#### Professionals facilitating perceived autonomy

##### Professionals’ activities created opportunities for perceived autonomy

Older people felt they had more autonomy if they received support from professionals.^22,28,60^ They said their autonomy was determined by the care atmosphere and whether they had the opportunities to influence decisions, express their will^20^ and make their own choices.^29^

Older people said that professionals respected their autonomy,^32,34,45,49,59^ and supported it,^54^ although respect varied among professionals and some showed very little.^32,54^ Respect for older people included acknowledging their choices,^54^ showing good manners, such as knocking on their bedroom door^49^ and treating them with dignity. It also included observing the need for privacy while conducting care practices.^50^ Older people felt that professionals lacked respect when they did not respond to their requests for help^32,45^ or made them wait unnecessarily.^32^

Studies showed that older people felt that autonomy was supported by recognising their will,^33,35,54^ although they wanted more opportunities to discuss what they wanted.^32^ However, older people acknowledged that respecting their will did not mean that professionals would always respect their wishes.^33^ Some older people said that their wishes were ignored,^57^ but that professionals rarely made decisions that were totally against their will.^29,65^ Some of the older people had made advanced directives and care plans to ensure that their requests were respected.^31,33^ However, others said that advance directives were useless, because, at the end of the day, professionals would decide whether they followed them.^33^

Older people felt that their autonomy could be supported by giving them information, which enabled them to improve how they controlled their decisions.^46^ However, the information they received was often insufficient.^20,32,46^ Those with higher levels of education said that they had received more information than those with lower educational levels,^24^ long-term illnesses, people with no relatives or those who rarely required nursing interventions.^25^

##### Professionals controlling choices

Older people talked about their perceived autonomy in relation to situations where professionals made decisions on behalf of them.^25–27,32,36,37,50,55^ These included what clothes to wear,^26,27^ the quality of the food they were given,^32,54^ when they should rest^32,50,54^ and have showers or baths, ^26,50,54^ meeting roommates^32^ or taking medication.^20,24,51,58^ Some older people said that the professionals should decide, because they knew what was best for them,^37,51^ even if they acted against their will.^51^ Some older people felt they were incapable of making their own decisions^26^ and some felt that professionals were responsible for their well-being^30^ and they trusted them to evaluate situations.^51^

However, some older people felt that their autonomy was constrained by the unethical conduct^54^ and coercion^49^ of professionals and that they had no choice. Older people’s views varied from accepting coercion to opposing it to some extent. Coercion was accepted in some cases, when older people felt that they did not know what was best for them, they were in danger of harming themselves or others or they needed help to protect their self-image and dignity. The respondents did say that if coercion was used, it should be carried out gently.^51^

Older people perceived that their opportunity to complain in care homes improved their autonomy. If older people were afraid of the potential consequences of any complaints,^49,54^ or did not want to appear troublesome,^32,46,54^ they felt they lost their autonomy.^59^ Other older people said they believed that complaining to professionals would change nothing, because they dismissed their opinions.^32,50^ Older people said that they promoted their perceived autonomy by getting along with professionals, following their requests and being polite.^54^

##### Enabling care practices

Older people said that care routines in residential care risked their autonomy.^26,32,49^ Strict daily routines decreased their control over their own lives and their ability to make decisions.^21,26,27,36,59^ For example, they were expected to attend some social activities, and had no chance to refuse,^26^ or they faced restrictions with regard to eating or washing.^38^ In contrast, others described how care routines encouraged them to do things by themselves^38^ and that steps had been taken to decrease the obvious safety risks.^26^

Older people felt that their autonomy was linked to the availability of assistance^44,49^ from relatives, friends or care professionals.^44^ They felt that insufficient^32,49^ or delayed help hindered their autonomy.^54,59^ These could partly result from older people refusing to ask for help^32,40,54^ or finding it difficult to ask for it.^44,46,50^

##### Professionals’ characteristics

Well-educated professionals enhanced older people’s perceived autonomy.^32,45,54^ In addition, professionals who were flexible in their working methods^38,54^ and had equal, positive^54^ and friendly attitudes towards older people supported their autonomy.^38^ Older people said it was important that professionals knew them and their needs.^59^ Their autonomy was hindered if professionals did not take the initiative to help them and if they felt that professionals displayed arbitrary, manipulative or power-seeking attitudes against older people.^54^

#### Living environments create opportunities for perceived autonomy

##### Organisational characteristics

The residential care environment they lived in was linked to older people’s autonomy.^56,57^ Their autonomy was greater if care homes involved older people in their individual care plans, let them decorate their own rooms and listened to their feedback on menus.^20^ People who had more influence on the organisation of their care home also had more positive perceptions of their autonomy.^29^ In contrast, older people felt that their autonomy was lower if they were restricted by group focused rules^57^ and had no chance to influence organisational matters.^54^

Older people felt that the type and size of the care facility influenced their autonomy. Older people’s autonomy was better recognised in residential care homes than in nursing homes^26^ or homes run by private organisations.^26,27^ In addition, older people living in smaller homes had more opportunities to make their own choices than those who lived in bigger units.^36^ Older people also said that political decisions that reduced residential care funding hindered their opportunities for perceived autonomy and their ability to receive the help they needed.^32,54^

Older people reported that limited resources in residential care homes risked their autonomy. Insufficient staffing and time constraints during sifts^26,32,45,49,50,54^ meant they had fewer opportunities to talk to care professionals and this resulted in limited autonomy.^32^ Insufficient staffing meant that older people went to bed earlier,^49^ had to wait for help when they needed to go to the toilet^32,54^ and faced restrictions on going outside.^32^ Organisational staff limitations meant that older people had fewer choices.^26^ Older people said that constantly changing care professionals and no opportunity to choose their caregivers risked their autonomy.^59^

##### Physical care facilitators

Older people felt that their autonomy was enhanced if they lived in an easily accessible care environment. They felt they had greater autonomy when they were able to move about freely if they needed a wheelchair and if doors were easy to open.^20,37,40,54,57,59,62^ Locked doors made older people feel that their autonomy was restricted,^59^ but they realised that such measures protected the safety of some residents and enabled others to have privacy.^49,59,62^

##### Social care facilitators

Privacy played a crucial role in older people’s perceived autonomy,^62^ although it was described as rare. This was due to limited access to private rooms, lack of privacy when they were making phone calls and no secure place for personal belongings.^20^ Older people who had their own room had more opportunities for privacy and to make their own choices than if they lived with others.^26^ Sharing a room with others could prevent older people from making own decisions about their treatment,^24^ when they had the lights on or off, and whether they could open windows for fresh air.^59^ Other residents could exhibit disturbing behaviour that hindered older people’s perceived autonomy.^54^ For example, during meal times, the loudest residents were served first, while others had to wait.^46^

Older people said that taking part in social activities had an impact on their perceived autonomy^32,36,46,54,61^ and the sense of control they had over their own lives.^61^ Social activities referred to parties and social gatherings,^36^ such as bingo, using a gym and other group activities,^46^ which were generally controlled by care professionals.^36,46^ Older people had limited chances to influence the content of the activities and choose which ones they took part in.^32,46^

The more visits from friends and relatives older people had, the more autonomy they felt they had.^22,23^ Even short visits could be seen as difficult or unacceptable, as they got in the way of the routines in the care homes. In addition, some professionals discouraged visitors that they felt were a burden.^56^ Some older people were forbidden to live with their partner, due to residential care rules, which meant that their spouse had to live in another care home.^52^

## Discussion

Autonomy is a basic value for older people. Our review found that they reported individual experiences with regard to perceived autonomy that was associated with many different outcomes. Their autonomy was influenced by different healthcare activities, circumstances and situations and how professional carers responded to their daily needs.

This review revealed many factors that supported or hindered perceived autonomy, including those related to older people’s capacity, the professionals who cared for them and the environment they lived in. Factors related to perceived autonomy can be viewed in a continuum, as the presence or absence of those factors, such as care that was provided or neglected, supported or hindered older people’s autonomy. The studies that we reviewed provided multilevel and many-faceted descriptions of these factors.

### Older people’s contextual autonomy in residential care

Older people’s perceived autonomy was based on dignity and referred to their ability to make their own choices and act in accordance with their own will in relation to daily activities in residential care. Interestingly, their perceived autonomy focused mainly on daily activities, but neglected the big, but rare, decisions about end-of-life treatment or lifestyle choices.^11^ However, professionals may consider such daily ethical issues as secondary to big decisions,^11^ even though older people feel that their daily life is very important. In future, older people’s autonomy should be studied further and this should include decisions that they rarely need to make.

Older people recognised that the greatest threat to their perceived autonomy was their increased dependency on care professionals for their daily physical needs.^68^ It is worth noting that being dependent on others in this way did not stop older people’s inner freedom and ability to think.^59^ In addition to their increased need for care, other individual factors supported or hindered older people’s perceived autonomy. Some of these were stable and unchangeable, such as educational level, while others compensable, such as decreased physical abilities.

Perceived autonomy could be related to relationships, such relatives being involved in the older person’s care and the ethical conduct of the care professionals. This could vary depending on how able the older person was to make decisions at certain times and in certain situations. In some situations, older people preferred to make their own decisions, while at other times they welcome shared decision-making with others. Older people have also been reported to willingly delegate decision-making to relatives or professionals, as acknowledged in our results. Thus, professionals need to be sensitive and recognise that older people can behave differently when it comes to specific decision-making situations.^69^ They also need to be aware of how relatives influence how older people perceive their autonomy. Relatives can help older people to be autonomous in a number of ways, such as helping them to make their wishes heard. They can also make demands that go against the older people’s views. However, it is worth noting that different generations can have varying perceptions about what is needed and how to express and experience autonomy. This generation gap can create misunderstandings and conflicts in families, not to mention criticism in relation to care practices.

### Professionals’ care practices and preventing violations of autonomy

The role that professionals play in older people’s perceptions of autonomy is highlighted by this review. We found that older people’s perceived autonomy could be hindered by strict care practices or ideologies that meant they were treated as subjects by care professionals. Care practices that meant that older people had to ask for help could also hinder perceived autonomy. This was because older people felt that it made them look weak^70^ or they didn’t want to be an extra burden to others. This sometimes led to some older people adopting a passive client role as they waited for the professionals to take care of them.^68^ In order to maintain their perceived autonomy and capacity for independence, older people may require assistance or need other people to advocate on their behalf so that their existing capacities are not reduced.

Perceived autonomy can be restricted in residential care due to safety issues that inhibit older people’s decision-making, such as whether they are allowed to get out of bed^68^ or move freely around the facility. These restrictions can be understandable when older people’s well-being or dignity is in danger. In these situations, care professionals should discuss these issues with older people and, or, their relatives so that they can come up with the best solutions to protect the older person’s autonomy, safety and well-being.

However, it is common for older people to experience mistreatment and violation of their autonomy and studies have shown that these are partly the result of professional practice.^71,72^ Mistreatment includes psychological abuse by restricting decision-making,^73^ ignoring older people’s preferences and coercing them into following instructions from professionals.^74^ The psychological abuse of older people was reflected in our results. There is limited research evidence to support the development of best practice,^75^ and further studies are needed to identify how to prevent violating older people’s autonomy. For example, intervention studies could help to identify professional practices that support older people’s autonomy. It is evident that organisational ethics and leadership, as well as professionals’ ethical competence and decision-making skills, need to be considered further.

### Strengths and limitations

This review had some strengths and limitations. The search phrases were formulated in collaboration with the library information specialist and research group to ensure their validity and to increase methodological rigour. The research group consisted of researchers with expertise in healthcare and ethics. The literature searches were conducted following a systematic search protocol. A limited number of databases were used, but they were the most suitable^76^ for the area covered by this review on how older people perceived their autonomy when living in residential care. A number of the papers we reviewed also included the views of professionals and relatives on older people’s autonomy, but these were not included as they lay outside the scope of this review. However, these views do warrant further investigation. Manual searches were also used to eliminate any search bias that may have resulted from the electronic searches, with regard to indexing, inconsistency in search terminology and variations in the term residential care. This additional search also maximised the number of relevant papers.^17^ We did not set any time limits on the start of the search, which was carried out in September 2019, and we identified papers going back to 1985. However, we did limit the search to peer-review journals published in English. The studies were independently selected by two researchers to support the validity of the papers that we included. Decisions about the inclusion criteria were based on the SPIDER tool, and the quality of the selected studies was evaluated using the MMAT. These were both designed to review both qualitative and quantitative studies.^18,19,77^

## Conclusion

This review provided new, synthesised knowledge on the perceived autonomy of older people living in residential care. Autonomy is a fundamental value in healthcare. In residential care, autonomy focused on older people’s decisions about their daily activities, but it included their dignity and human rights. Our review found that older people’s perceived autonomy was closely intertwined with their social relationships with relatives, professionals’ care practices and residential care environments. It is noteworthy that older people felt that autonomy promoted their health and quality of life, but that healthcare professionals also played a crucial role in facilitating and enabling their autonomy. Factors that supported and inhibited older people’s perceived autonomy in residential care had already been well identified by a number of studies. The practical implications of the study findings are that older people’s perceived autonomy needs to be acknowledged throughout everyday life and these can range from small daily issues to rare decisions. In addition, older people need to be supported so that they can exercise their autonomy and they should receive individual support that respects their wishes and needs. The knowledge that this review provides can be used to plan, implement and evaluate care in residential facilities. In future, more attention needs to be focused on how older people’s perceived autonomy in residential care is affected by professionals’ ethical competencies, leadership and care practices and the environment that older people live in.
